# On Training Neural Network Decoders of Rate Compatible Polar Codes via Transfer Learning

**DOI:** 10.3390/e22050496

**Published:** 2020-04-25

**Authors:** Hyunjae Lee, Eun Young Seo, Hyosang Ju, Sang-Hyo Kim

**Affiliations:** 1 Department of Electrical and Computer Engineering, Sungkyunkwan University, Suwon 16419, Korea; 2 Samsung Electronics, Hwaseong 18448, Korea

**Keywords:** polar codes, deep learning, neural network decoder, transfer learning

## Abstract

Neural network decoders (NNDs) for rate-compatible polar codes are studied in this paper. We consider a family of rate-compatible polar codes which are constructed from a single polar coding sequence as defined by 5G new radios. We propose a transfer learning technique for training multiple NNDs of the rate-compatible polar codes utilizing their inclusion property. The trained NND for a low rate code is taken as the initial state of NND training for the next smallest rate code. The proposed method provides quicker training as compared to separate learning of the NNDs according to numerical results. We additionally show that an underfitting problem of NND training due to low model complexity can be solved by transfer learning techniques.

## 1. Introduction

Polar codes, proposed by Arikan in [1], are the first error correcting codes to provably achieve the symmetric capacity with low complexity in binary-input discrete memoryless channels (B-DMCs). This result means that Shannon’s random codes, which achieve channel capacity, are replaced by a practical code with a low-complexity decoding algorithm [1]. Due to its better performance compared to turbo and low-density parity-check (LDPC) codes at short lengths, it has been adopted as the error correcting code for control signals in the enhanced mobile broadband (eMBB) scenario of the third generation partnership project (3GPP) 5G standard [2].

Decoding schemes of polar codes are commonly used with successive cancellation (SC) [1] and belief propagation (BP) algorithms [1]. SC decoding has a relatively low computational complexity and high latency, while BP decoding has a high throughput and computational complexity [3]. In addition, both schemes perform poorly at finite lengths, e.g., hundreds to thousands of bits. SC list (SCL) decoding with concatenated a cyclic redundancy check (CRC) code was proposed later [4], and it was proved that similar performance can be obtained as compared to turbo and LDPC codes in finite lengths. That is, polar codes have been shown to be practically available. Recently, decoders using deep learning (DL) have been proposed to replace traditional decoders of polar codes [5,6,7,8,9,10,11].

DL has made a great success in computer vision [12], machine translation [13], speech and image recognition [14], and many other fields. Its influence has become widespread and has reached communication systems, where analytic solutions have been preferred. There have been a number of DL approaches [5,15,16] to physical layer communications. Among them, we have got a special interest in deep neural networks (DNN)-based decoding of channel codes. As a pioneering work, Gruber et al. employed feedforward deep neural networks to learn the maximum a posteriori (MAP) decoding of polar codes [5]. That approach was extended to convolutional neural networks (CNN) and recurrent neural networks (RNN) and it was shown that better performance can be obtained by the advanced structures of DNN [6]. Such small neural network decoders (NNDs) are used to form a decoder for longer polar codes in combination with BP processing [7]. Nachmani et al. designed an NND called the ‘neural BP decoder’ that basically performs weighted BP decoding with variable weights [8]. A proper training finds the weight values that compensate finite length impairments of the BP algorithm and the performance is therefore improved for high density parity check codes such as Bose, Chaudhuri, and Hocquenghem (BCH) codes. Bennatan et al. proposed a syndrome-based NND for linear block codes [9]. For CRC-polar codes, a neural BP algorithm was proposed by Doan et al. [10]. In [17], Tandler et al. proposed an ordering of data for efficient training of the decoder of convolutional codes. Unlike the problems of applying general deep learning, NND has an advantage that it is very easy to generate a training data set. In addition, the NNDs are capable of one-shot decoding because the received signal is decoded only once through the hidden layers, and can approach the optimal decoding performance with low latency.

Previous studies [5,6,7,8,9,10,11,17] considered learning an NND for a specific code of fixed length and rate. However, wireless standards normally use a class of codes of multiple parameter sets, since a receiver is required to have either multiple decoders each of which is specialized to a code or a decoder which is flexible to decode many codes. For NND, a decoder is determined by the values of the weights. To support multiple codes, the same number of sets of weight values should be stored even though the NND hardware is commonly used. Because the supervised learning method [5,6] trains a decoder with data from a specific code and channel, a straightforward approach is to train all the decoders separately. However, we thought if codes are closely related, then decoder training can be aided by the results of the training of another decoder.

In this paper, we consider the problem of training a set of rate-compatible polar codes that are expurgated from the same mother code. Exploiting the inclusion property of rate-compatible polar codes which are defined by a single polar coding sequence as adopted in a 5G New Radio (NR) [2], we propose an efficient decoder training method via transfer learning. We compare the complexity and performance of the proposed method with conventional separate learning. We also tackle an underfitting problem where the given model complexity is not sufficiently high to be well trained by conventional training methods. It is shown that transfer learning from low to high-rate codes can train a high-rate code decoder better than the conventional training methods.

The rest of this paper is organized as follows. In Section 2, we briefly introduce the NND framework, basics of polar codes, and the conventional NND for polar codes. The characteristics of rate-compatible polar codes and their training data are investigated and the proposed transfer learning-based training method of NNDs for the rate-compatible polar codes is then presented in Section 3. Performance comparison with the conventional separate learning is given in Section 4. Section 5 concludes the paper.

## 2. DL Based Decoders for Polar Codes

In this section, we introduce the conventional decoding of polar codes and the system framework of NND for polar codes. We consider an implementation of an NND of polar codes based on the work in [6].

### 2.1. System Framework

**Figure 1:**
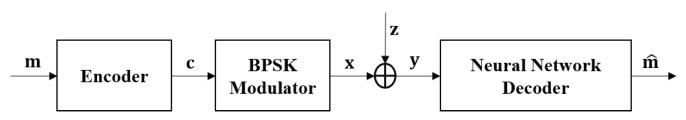
System model with a neural network decoder.

Artificial neural networks are a network of many artificial neurons that pass signals with variable weights so as to approximate an arbitrary nonlinear function. The weights for the signals can be trained by a set of data to mimic a desired function that minimizes a cost function [18]. In this paper, we consider the problem of training NND of rate-compatible polar codes based on the previous setting in [6]. Our system model that comprises an NND is depicted in Figure 1. Let *N*, *K*, and *R* be the code length, message size and code rate, respectively and then R=K/N. At the transmitter, a message $m=\left(m_{1},\ldots ,m_{K}\right)\in {\left\{0,1\right\}}^{K}$ is encoded to a codeword $c=\left(c_{1},\ldots ,c_{N}\right)\in {\left\{0,1\right\}}^{N}$. Then, c is modulated by the binary phase shift keying (BPSK) modulation and the transmitted signal $x=(x_{1},x_{2},\ldots ,x_{N})$ with $x_{i}={\left(-1\right)}^{c_{i}}$, 1≤i≤N, is sent through the additive white Gaussian noise (AWGN) channel. At the receiver, $y=(y_{1},\ldots ,y_{N})=x+z$ is received, where $z=(z_{1},\ldots ,z_{N})$ is the Gaussian noise vector with zero mean and variance $\sigma^{2}$. Since $E[x^{2}]=1$, the noise variance is determined as $\sigma^{2}=\frac{1}{2R}\frac{1}{\left(E_{b}/N_{0}\right)}$ for a given $E_{b}/N_{0}$ value. Then, the NND outputs the estimated message $\hat{m}=\left({\hat{m}}_{1},\ldots ,{\hat{m}}_{K}\right)$ by decoding y.

### 2.2. Polar Codes

Polar codes are the first family of provably capacity-achieving codes with low decoding complexity in B-DMCs based on the channel polarization phenomenon [1]. Channel polarization consists of two phases: channel combining and channel splitting. Through this process, *N* independent copies of a channel *W* having the same channel capacity are transformed to *N* synthetic split channels with different channel capacities. The capacity polarizes to either 0 or 1 as *N* goes to a very high value. Let $S_{\alpha }$ be the sequence of the indices of split channels sorted in the descending order of capacity for a finite *N*, where α denotes the parameter of the channel. A polar code of dimension *K* is defined by the index set $I_{K}$ which is composed of the best *K* channels, the elements of $S_{\alpha }\left[1:K\right]$. The set $I_{K}$ is said to be the ‘information set’ and $I_{K}^{c}=\left\{1,2,\ldots ,N\right\}\backslash I_{K}$ is called the ‘frozen set’ whose corresponding bits are preset with known values, e.g., zero. The source vector $u=\left(u_{1},\ldots ,u_{N}\right)\in {\left\{0,1\right\}}^{N}$ is formed by the subvectors, $u_{I_{K}}$ = m and frozen $u_{I_{K}^{c}}$. The codeword c is encoded by $c=uG_{N}$, where the generator matrix $G_{N}$ is defined by $G_{N}=F^{⊗n}$, $F=\left[\begin{array}{cc} 1 & 0\\ 1 & 1 \end{array}\right]$, and ${}^{⊗n}$ denotes the *n*-th Kronecker power.

Polar codes achieve capacity asymptotically under SC decoding [1]. However, its finite length performance was not impressive in comparison to turbo or LDPC codes. SCL decoder was later proposed for better performance in finite length [3,4]. SCL decoding chooses the best of *L* surviving decoding paths whereas SC decoding maintains only one path during a decoding. SCL decoding converges to the MAP decoding as *L* increases. However, it is hard to fully parallelize the entire decoding procedure due to the sequential nature of the decoding algorithm.

### 2.3. NNDs for Polar Codes

Since NNDs can show near MAP performance at extremely low latency for short codes, they have been studied as a new solution to low latency applications for polar codes [5,6,7,8,9,10,11]. In [5,6], it was shown that various neural networks are able to learn polar decoding algorithms for short codes and when fully learned, they perform closely to MAP decoding.

In this section, we review the system model, the neural network (NN) models for NNDs, and the training method of a single polar NND with a fixed rate proposed in [6]. We consider the transmission of BPSK modulated signals of polar code codewords over AWGN channels. We consider only multi-layer perceptron (MLP) and long short-term memory (LSTM) [19] models for NND structure. The NNDs are trained via supervised learning.

Supervised learning is one of the training methods of DL, which uses training data consisting of inputs and desired labels for those inputs where the decoding function is regarded as a multi-class classification. The training data set is a set of data points, each of which consists of y and m; an input and its label. Unlike many typical supervised learning problems, data can be collected as much as desired since the system model is a natural data generator of this problem. First, m is randomly selected from $2^{K}$ messages and is encoded, modulated, and transmitted through the AWGN channel in the probabilistic model. The received vector y and the corresponding message m form a training data point. First, the MLP model under consideration is introduced. The MLP is a fully connected feed-forward NN model which is composed of the input and output layers along with several hidden layers. For MLP, we employ the same structure as in [6]. The numbers of the nodes of the input and output layers are *N* and *K*, respectively. We only consider polar codes of lengths 16 and 32 because it is hard to learn a long block code decoder by this general NNDs. Three hidden layers of 128-64-32 nodes are commonly used. The rectified linear unit (ReLU) is used in hidden layers and the sigmoid function is adopted for the output layer. We also use the LSTM model based on the structure proposed in [6]. A single LSTM cell is used for the LSTM model in this paper. The output dimension of the LSTM cell is 256 and each node uses the sigmoid function. The input vector is sequentially fed symbol by symbol. After *N* time steps, *K* nodes are selected as the output nodes from the 256 nodes.

Training is conducted so as to minimize the average loss. As loss function, we use the mean squared error (MSE) defined as

(1) $$L=\frac{1}{K}\sum_{i=1}^{K}{\left(m_{i}-{\hat{m}}_{i}\right)}^{2},$$

where $m_{i}$ and ${\hat{m}}_{i}$ are symbols of the label and the output, respectively. Weights and biases are updated via stochastic gradient descent (SGD) in batches. Because of the characteristics of the system model, noisy transmission can take on infinitely many values. This makes the concept of ‘epoch’ obscure, because an ‘epoch’ originally indicates a single round of training with an entire finite data set. In [5], the pair of a message m and its modulated codeword x was considered as a data point. The size of data set is then $2^{K}$ and one data point exists for each class of the decoding problem. To reflect the noisy transmission, a noise layer was included at the input of the NN model to be trained. Training through the entire set of code was counted as a single epoch. On the other hand, in [6], the noisy received vector y is paired with its message m to form a data point. A set of data points of a fixed size was used as a batch for parameter update. As random noise is added in each transmission, data points can be generated unlimitedly, which can make the definition of epoch pointless. However, the weight update with a batch was interpreted as an epoch in [6].

In this paper, we follow the interpretation of [6] for the definition of a data point. However, the concept of epoch is ignored because the size of available data can be infinite. Instead, we evaluate the complexity and speed of training in the number of mini-batches of a fixed size $l_{b}$. The number of mini-batches or weight updates in a training session is denoted by $M_{b}$.

The eventual goal of the decoder training is obviously to obtain a decoder performing close to the MAP decoder. The trained NND is evaluated in terms of bit error rate (BER) between m and $\hat{m}$ via Monte Carlo simulation.

## 3. Training of NNDs via Transfer Learning

Since the decoding function for the NND [5,6] is a classification into all codewords, the number of classes (i.e., the size of the code) grows exponentially with the code dimension *K*. Naturally, the training data should be sufficiently larger than the number of classes. Therefore, the training complexity is a major bottleneck for a long code although the generation of a data set is easy. This limits the training problem to only codes of a small *K*. Even though the code is short, training complexity increases when we need to support rate-compatible codes.

If a set of polar codes is constructed based on expurgation that uses a single polar coding sequence [2], a low rate code is included in a higher rate code within the family of codes. In this section, we introduce a method of generating training data using the inclusion relationship between codewords according to the code rate of polar codes. Then, we propose an efficient training method for the NNDs of multiple polar codes in the context of transfer learning. We also suggest to train a single decoder via transfer learning method to solve an underfitting problem due to low model complexity.

### 3.1. Inclusion Relation of Training Data

In this subsection, we investigate the inclusion relation of the polar codes defined by a single polar coding sequence. To support rate-compatibility of polar codes, multiple codes with rate $R=\{R_{i}=K_{i}/N\},\;1\leq i\leq T$ should be used. Assume without loss of generality $R_{i}<R_{j}$ for i<j. Because the polarization is channel-sensitive, the optimal construction for $R_{i}$ requires the optimal channel parameter $\alpha_{i}$ and the corresponding order $S_{\alpha_{i}}$. However, to reduce the complexity of description, a unified sequence *S* can be used for multiple rate-compatible codes with small performance penalty as adopted in 5G NR [2]. Let us define such a set of codes as follows.

**Definition** **1.** *Let $C=\{C_{i}\}$, 1≤i≤T be a rate-compatible set of polar codes supporting R where $C_{i}=C\left(N,K_{i}\right)$ and $C(N,K_{i})$ is the polar code of dimension $K_{i}$ defined by the unified polar coding sequence S.*

It is manifest that the inclusion relation $I_{K_{i}}\subset I_{K_{j}}$ for i<j holds because $I_{K}=S\left[1:K\right]$ for rate-compatible polar codes based on a single polar coding sequence. Let $G_{I_{K_{i}}}$ be the submatrix of *G*.

**Proposition** **1.** *Polar code $C_{i}$ is linear if the frozen vector $u_{I_{K_{i}}^{c}}=0$.*

**Proof.**  Assume $u_{I_{K_{i}}^{c}}=0$ and for $c^{\left(i\right)}\in C_{i}$,

$$\begin{array}{cc} c^{\left(i\right)} & =uG\\ & =u_{I_{K_{i}}}G_{I_{K_{i}}}+u_{I_{K_{i}}^{c}}G_{I_{K_{i}}^{c}}\\ & =u_{I_{K_{i}}}G_{I_{K_{i}}}, \end{array}$$

which proves that $C_{i}$ is a binary linear code with the generator matrix $G_{I_{K_{i}}}$. □

**Proposition** **2.** *Assume all $C_{i}\in C,1\leq i\leq T$, are linear by setting $u_{I_{K_{i}}^{c}}=0$. For i<j, we have $C_{i}\subset C_{j}$.*

**Proof.**  For a codeword $c^{\left(j\right)}\in C_{j}$

(2) $$\begin{array}{cc} c^{\left(j\right)} & =u_{I_{K_{j}}}G_{I_{K_{j}}}+u_{I_{K_{j}}^{c}}G_{I_{K_{j}}^{c}}\\ & =u_{I_{K_{j}}}G_{I_{K_{j}}}\\ & =u_{I_{K_{i}}}G_{I_{K_{i}}}+u_{I_{K_{j}}\backslash I_{K_{i}}}G_{I_{K_{j}}\backslash I_{K_{i}}}, \end{array}$$

since $u_{I_{K_{j}}^{c}}=0$. Due to (2) for a codeword $c^{\left(i\right)}\in C_{i}$,

$$\begin{array}{cc} c^{\left(i\right)} & =u_{I_{K_{i}}}G_{I_{K_{i}}}+0G_{I_{K_{j}}\backslash I_{K_{i}}}\\ & =\left([u_{I_{K_{i}}}0]P\right)G_{I_{K_{j}}}\in C_{j}, \end{array}$$

where 0 is the zero vector of length $K_{j}-K_{i}$ and there exists a permutation matrix *P* satisfying the last equality. It has been proved that codeword $c^{\left(i\right)}$ corresponding to message $u_{I_{K_{i}}}$ is the codeword of $C_{j}$ for message $[u_{I_{K_{i}}}0]P$. □

**Figure 2:**
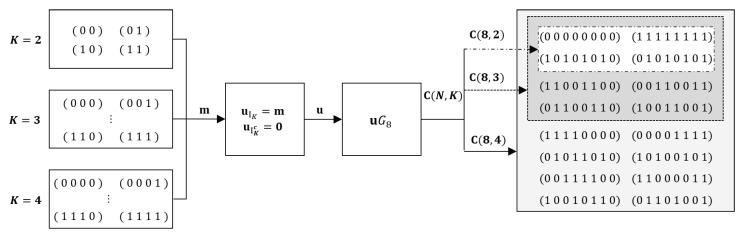
Sets of messages and corresponding codes, C(8,2),C(8,3) and C(8,4) and their inclusion relationship.

Figure 2 exhibits an example of the inclusion relationship of polar codes C(8,2),C(8,3), and C(8,4) with generator matrix $G_{8}=F^{⊗3}$ and S=(8,7,6,4,5,3,2,1). Note that C(8,2)⊂C(8,3)⊂C(8,4). Since $C_{i}\subset C_{j}$ holds for i<j and $I_{K_{i}}\subset I_{K_{j}}$, data points generated for the code of dimension $K_{i}$ can be valid data points for the code of $K_{j}$. The set of training data for C can be made to have an inclusion relation between the data for individual codes. The data used to train the NND for $C_{i}$ is a valid subset of data for training the NND for $C_{j}$. Therefore, we apply transfer learning to train an NND for $C_{i+1}$ by adopting the NND trained for $C_{i}$ as the initial state. Transfer learning can be applied recursively to the sequence of NND training in the increasing order of rate. In the next subsection, we describe the procedure of transfer learning for training NNDs of rate-compatible polar codes in detail.

### 3.2. Transfer Learning for NNDs of Rate Compatible Polar Codes

Our problem is to train |C|=M NNDs where the elements in C are equally long rate-compatible polar codes as defined in Section 3.1. A naive approach is to train them independently, but a more efficient way can be considered. Complexity of NND training is counted in the number of mini-batches. Let $M_{b}^{tot}=\sum_{i}^{T}M_{b}^{\left(i\right)}$ be the total complexity, where $M_{b}^{\left(i\right)}$ is the complexity used for training the $C_{i}$-NND. We set the size of the mini-batch $l_{b}$ to 128 throughout the paper. We consider the application of transfer learning [20,21] for decoder training, which has been used when similar problems and solutions exist on an NN model, where a trained model can be reused to boost the training of another problem. For a given $M_{b}^{tot}$, we pursue a more efficient training in terms of performance of the NNDs.

As noted in the previous section, each data point used to train the decoder for $C_{i}$ is a valid data point for $C_{i+1}$. So we assume that the decoder for $C_{i}$ may be a good initial state of the training phase of $C_{i+1}$. In other words, the learned state of an NND is transferred to the NND for a code of a higher dimension at the beginning of a training session. If transfer learning is effective, the overall complexity of the training may be reduced by this approach. In order to reduce the complexity, the training should be planned well. We train the NNDs in increasing order of rate. So we start from the code of the lowest rate and the training procedure is described in detail below.

In Algorithm 1, the training procedure of NNDs via transfer learning is described. NNDs are trained in increasing order of rate using a single NN model. The NN model used is either the MLP or the LSTM described in Section 2.3. The NN model is initialized with random weights first. Let $C_{i}$-NND be the decoder for code $C_{i}$. The training data is generated for training of $C_{i}$-NND and the decoder is then trained $M_{b}^{\left(i\right)}$ mini-batches. How to generate data is described in Algorithm 2. The learned state of $C_{i}$-NND is stored and transferred to the training of $C_{i+1}$-NND as its initial state. MSE is used for the cost function and SGD is used as the optimizer. For the training of a low rate code NND where $K_{i}<K_{T}$, redundant output nodes are labeled as 0.5 which remains neutral between 0 and 1. This procedure is repeated until the last decoder, $C_{T}$-NND training is finished. The entire procedure can viewed as a multi-step training of an NN toward a good $C_{T}$-NND, during that the state of NN is sampled as $C_{i}$-NND.

Each NND can be tested concurrently along with its training. A test evaluates the BER of the decoder where a bit error is counted when the message mismatches with the output truncated to the message length $K_{i}$. When data is generated, the CollectData(·) function defined in Algorithm 2 collects $N_{sample}$ data points each of which is a pair of a message m and a received signal y or the output of an AWGN channel. A message is randomly generated from the entire set of messages. The message is encoded by $C_{i}$ and modulated with BPSK, and then sent through an AWGN channel where a Gaussian noise vector z is added to the transmitted vector x. Random sampling of the received vector is repeated without replacement $N_{sample}$ times. For the AWGN channel, the channel parameter $E_{b}/N_{0}$ is chosen empirically.
**Algorithm 1** Train NNDs via transfer learning for rate-compatible polar codes.**Input:** Rate R=($R_{1},\ldots ,R_{T})$, code length *N*, numbers of mini-batches $M_{b}^{\left(i\right)}$’s, polar coding sequence *S*
  1:Initialize the $C_{1}$-NND with random weights   2:**for***i* = 1 : *T* **do**  3: $X_{i}\leftarrow \mathrm{CollectData}\;(i)$  4: Train $C_{i}$-NND with $M_{b}^{\left(i\right)}$ mini-batches random-sampled from $X_{i}$    5: Store $C_{i}$-NND    6: **if** *i* < *T* **then**  7:  Initialize $C_{i+1}$-NND with $C_{i}$-NND    8: **end if**   9:**end for**10: **return** All NNDs  


**Algorithm 2**CollectData(i).**Input:** index *i*, code length *N*, polar coding sequence *S*, training $E_{b}/N_{0}$, data size $N_{sample}$
  1:Generate all messages $M=(m_{1},\ldots ,m_{2^{K_{i}}})$ of length $K_{i}$  2:Empty data set X    3:**for**$j=1:N_{sample}$**do**  4: Initialize u as the zero vector    5: Select a message $m^{\prime }$ randomly from M    6: Determine the information and frozen vectors ($I_{k}\leftarrow S\left[1:K\right]$ and $u_{I_{K}}\leftarrow m‘$)    7: Make the transmitted signal ($c\leftarrow uG_{N}$, $x\leftarrow (x_{1},x_{2},\ldots ,x_{N})$ with $x_{k}={\left(-1\right)}^{c_{k}}$)    8: Channel operation (y←x+z where z is iid Gaussian with variance $\sigma^{2}=\frac{1}{2R}\frac{1}{\left(E_{b}/N_{0}\right)}$)  9: Add $X_{j}=\left(y,m^{\prime }\right)$ to X10: **end for**11: **return**X


The benefit of transfer learning lies in the efficiency of training. In order to get a well trained set of NNDs, the total complexity $M_{b}^{tot}$ should be properly distributed among the $M_{b}^{\left(i\right)}$, i=1,…,T. Faster learning due to transfer learning saves training complexity for small *K* so that NNDs for a larger *K* can be trained more. To show the advantage of the proposed learning method effectively, the uniform allocation $M_{b}^{\left(i\right)}=M_{b}$, is considered for the conventional separate learning.

### 3.3. Training of Individual NND via Transfer Learning

In this subsection, we consider the training problem of a single NND with a limited model. According to previous results [5], polar NNDs have been well trained from the MLP model when *N* is 16 or smaller. Similar performance was achieved on LSTM models with a lower model complexity but higher training complexity [6]. Assume we want to train a $C_{k}$-NND individually from an NN model. If the model complexity is not sufficiently high, the model might underfit even though the data size and $M_{b}$ are large. We propose to use transfer learning to solve the underfitting problem. It will be shown that multi-step training with a proper sequential application of data sets can train the $C_{k}$-NND better at the same complexity. We simply run Algorithm 1 with a given total training complexity $M_{b}^{tot}$. However, training data $X_{i}$’s and $M_{b}^{\left(i\right)}$ are sequentially applied from a certain value of i<k.

## 4. Numerical Results

In this section, we numerically evaluate our proposed transfer learning technique for rate-compatible polar codes. The training results are compared with those of separate learning in terms of performance. We assume the BPSK modulation and the AWGN channel for all simulations. Codes with parameters (N,K)=(16,3−8) and (32,7−16) were used. The polar coding sequence defined in [2] was used in the construction of such codes. As mentioned an MLP and an LSTM model is used for the corresponding NNDs. The structures of the NN models were specified in Section 2.3. The detailed parameter setting is shown in Table 1. A 64-32-16 MLP and an LSTM have similar complexities in terms of the number of trainable parameters. For the training, the dropout and learning rate are set to 0.1 and 0.0009, respectively. As noted, MSE is used for the loss function as in Section 2. The SGD method with ADAM optimizer [22] is used. The training is implemented using TensorFlow. The hyper-parameters of NN training are listed in Table 2. Polar codes of parameter sets (N,K)=(16,3−8), (32,7−12), and (32,11−16) are used for training multiple MLP and LSTM-based NNDs.

We generate $N_{sample}=10^{6}$ training data points for each training session. The training data of the proposed method is generated at $E_{b}/N_{0}=4\;\mathrm{dB}$ according to Algorithm 2. We did not rigorously optimize the training $E_{b}/N_{0}$ to simplify comparison. On the other hand, the separate learning generates data with the training ratio *p* [6], which is the portion of codewords used to generate training data, compared to the entire code. In this simulation, we set p=(0.4,0.6,0.8,1.0). That is, the selecting from all messages in Algorithm 2 is changed from the selecting a smaller message set to *p*. For each *p*, we took 5 different training $E_{b}/N_{0}$ points from −2.0 to 6.0 dB. We train the NND using the total of 20 training data and select the parameters that show the best test performance. We assume the complexity of training with a mini-batch of a fixed size is similar among the codes of the same length. To train the NNDs for a given set of rate-compatible codes, uniform allocation of complexity or the number of weight updates is considered. For both the proposed and separate learning methods, the numbers of weight updates are assigned as $M_{b}^{\left(i\right)}=5000$ for N=16 and $M_{b}^{\left(i\right)}=50,000$ for N=32. Note that we are interested in a training setting with constrained computing resources. The trained decoders are evaluated in terms of BER for the considered communication system. A test set has $10^{5}$ data points for each $E_{b}/N_{0}$ point ranging from 0 to 6 dB.

**Figure 3:**
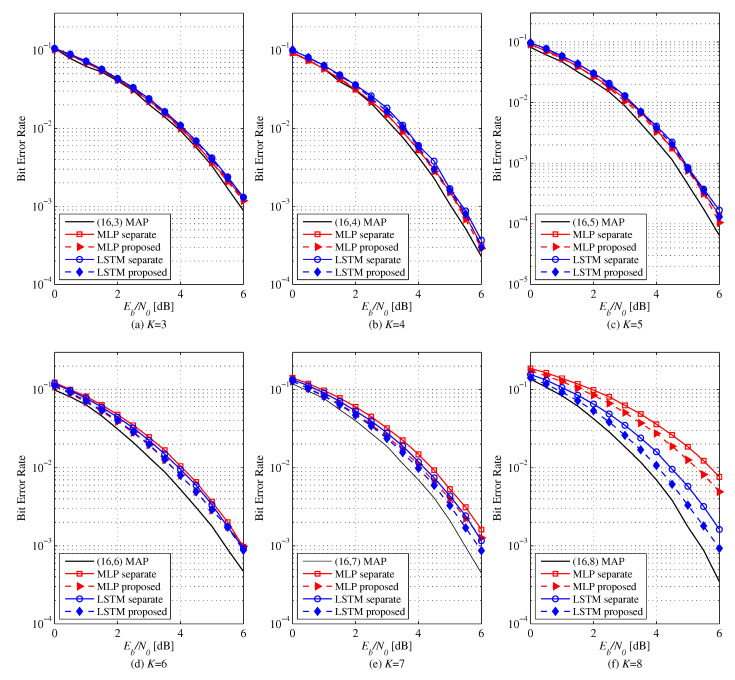
BER versus $E_{b}/N_{0}$ of MLP (128-64-32) and LSTM based NNDs for (N,K)=(16,3−8) polar codes. Comparison between the proposed and separate learning.

The NNDs trained by separate learning perform closely to MAP decoding for N=16 and all rates if $M_{b}^{\left(i\right)}$ are sufficiently large without training ratio adjustment (p=1.0). However, the proposed method trains the NNDs quicker. Figure 3 shows the BER of MLP and LSTM-based decoders for (N,K)=(16,3−8) polar codes. At low rates, both learning methods perform similarly. However, as the code rate increases, the proposed method shows better decoding performance than the separate leaning. Especially, when K=8, MLP attains a coding gain of 0.6 dB and LSTM gets 0.5 dB with the proposed learning method. Via transfer learning, good performance can be achieved with smaller $M_{b}^{\left(i\right)}$, i.e., less learning complexity, as the code rate increases. If $M_{b}^{\left(i\right)}$ is increased at all code rates, the decoding performances of all NNDs come close to MAP decoder.

**Figure 4:**
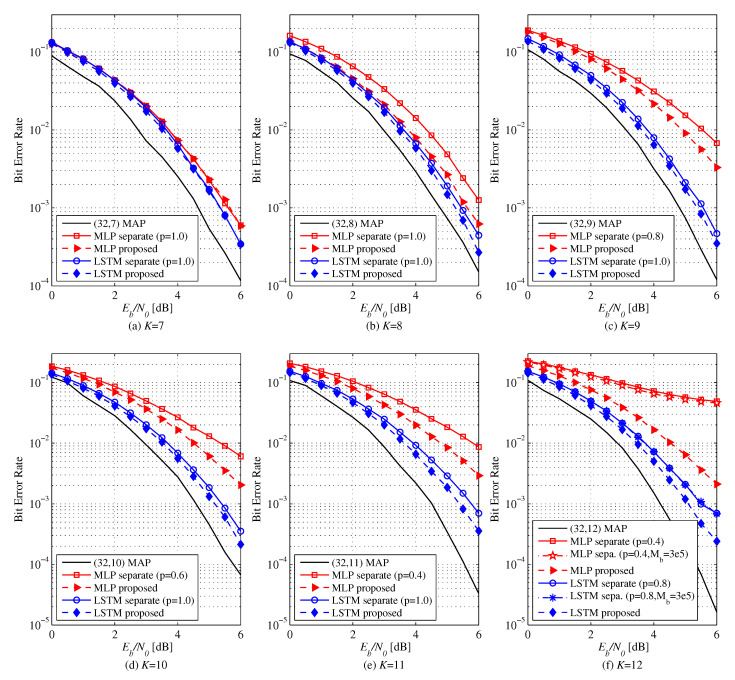
BER versus $E_{b}/N_{0}$ of MLP (128-64-32) and LSTM based NNDs for (N,K)=(32,7−12) polar codes: Comparison between the proposed and separate learning. Training ratio *p* is optimized to show the best performance. In (f), $M_{b}^{\left(i\right)}=50,000$ for all cases except for MLP and LSTM with separate learning for which $M_{b}^{\left(6\right)}=300,000$.

**Figure 5:**
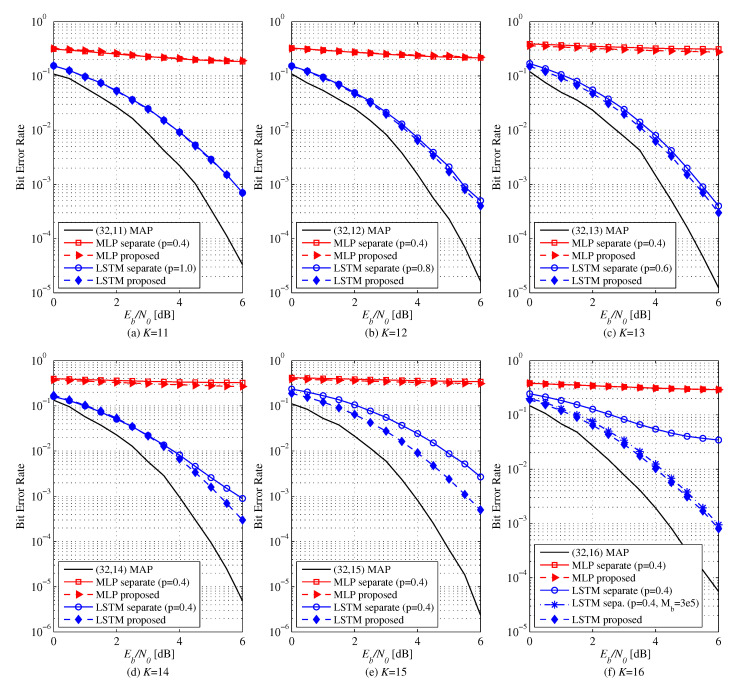
BER versus $E_{b}/N_{0}$ of MLP (64-32-16) and LSTM based NNDs for (N,K)=(32,11−16) polar codes: Comparison between the proposed and separate learning. Training ratio *p* is optimized to show the best performance. In (f), $M_{b}^{\left(i\right)}=50,000$ for all cases except for LSTM with separate learning for which $M_{b}^{\left(6\right)}=300,000$.

Figure 4 and Figure 5 show the BER performance of different learning methods for polar codes of N=32. Unlike the case of N=16, NNDs are not trained to achieve MAP performances. The BER of the MLP and LSTM-based decoders for (N,K)=(32,7−12) is shown in Figure 4. Separate learning can achieve a better performance with an adjustment of *p* down to 0.4. while the NND underfits for p=1.0. For low rates, performances are similar between the proposed and the separate learning methods as for N=16. However, as the code rate increases, the proposed method outperforms the conventional one. The MLP-based decoder with proposed method achieves a performance gain of 0.5 dB for K=8, 0.7 dB for K=9, 0.9 dB for K=10, and 1.0 dB for K=11 at a BER of $10^{-3}$ over the separate learning. For K=12, the separate learning fails to train the decoder well even for large $M_{b}^{\left(6\right)}=300,000$. On the other hand, the proposed method shows a much better error performance already at $M_{b}^{\left(6\right)}=50,000$. The performance gain of LSTM-based decoder with the proposed method is 0.2 dB for K=8, 0.2 dB for K=9, 0.3 dB for K=10, 0.5 dB for K=11, and 0.5 dB for K=12. For K=12, the performance of the separate learning does not improve as $M_{b}^{\left(6\right)}$ increases even to 300,000 from 50,000. As a result, we confirm that the proposed method mitigates the underfitting problem of the separate learning.

Figure 5 exhibits the BER performance of MLP and LSTM-based decoders for (N,K)=(32,11−16). We employ a smaller 64-32-16 MLP whose number of parameters is similar to that of the LSTM-based decoder with single cell of 256 units for fair comparison. For K≤12, there is no difference in performance of the LSTM-based decoder between the two learning methods. The proposed method performs better for K≥13. For K=16, separate learning does not train the LSTM-based decoder well at rather small $M_{b}^{\left(6\right)}=50,000$ although the performance eventually improves up to $M_{b}^{\left(6\right)}=300,000$. On the other hand, the proposed method trains the NND faster without showing error floor at $M_{b}^{\left(6\right)}=50,000$, already. It has been confirmed that NNDs can be trained by the proposed transfer learning method in a lower complexity than the separate learning method. As you can see, the MLP-based decoder does not learn at all for both the proposed and separate learning methods, whereas the LSTM-based decoder performs fairly well. It seems that the lower triangular structure of the generator matrix $G_{N}$ induces a desired but hidden sequential processing that is better learnable by the LSTM model under the model complexity constraint than the MLP model.

## 5. Conclusions

In this study, we proposed a method of training NNDs for a family of rate-compatible polar codes. It was indicated first that the inclusion property of rate-compatible polar codes allows the training of multiple corresponding NNDs to share data points so that transfer learning is possible. A training procedure of multiple NNDs via transfer learning was proposed and it was empirically verified that the proposed method speeds up the training of NNDs. When the model complexity is not sufficiently large, it was observed that even an underfitting problem can be solved by the multi-step transfer learning method. However, the proposed method has been verified only for very short codes. As future work, an extension of transfer learning to more advanced DL methods for longer rate-compatible codes can be studied. For instance, transfer learning technique can be applied to train the neural BP decoders [8] by gradually pruning check node neurons for augmented linear codes.

## Figures and Tables

**Table 1:** Total number of parameters in NN models.

*N*	*K*	MLP	LSTM	*N*	*K*	MLP	LSTM	*N*	*K*	MLP	LSTM
		(128-64-32)				(128-64-32)				(64-32-16)	
16	3	12,384	1796	32	7	14,560	2820	32	11	4784	3844
	4	12,416	2052		8	14,592	3076		12	4800	4100
	5	12,448	2308		9	14,624	3322		13	4816	4356
	6	12,480	2564		10	14,656	3588		14	4832	4612
	7	12,512	2820		11	14,688	3844		15	4848	4868
	8	12,544	3076		12	14,720	4100		16	4864	5124

**Table 2:** Hyper-parameters of NND training.

Size of training data per $E_{b}/N_{0}$	1,000,000
Training $E_{b}/N_{0}$ of separate learning [dB]	(−2.0, 0.0, 2.0, 4.0, 6.0)
Training $E_{b}/N_{0}$ of proposed learning [dB]	4.0
Size of test data per $E_{b}/N_{0}$	100,000
Test $E_{b}/N_{0}$ [dB]	(0.0, 0.5, *…*, 6.0)
Dropout probability	0.1
Learning rate	0.0009
Optimization method	Adam optimization
$l_{b}$	128
$M_{b}$	5000 (N=16),
	50,000 (N=32)
